# Low Sodium Intake, Low Protein Intake, and Excess Mortality in an Older Dutch General Population Cohort: Findings in the Prospective Lifelines-MINUTHE Study

**DOI:** 10.3390/nu15020428

**Published:** 2023-01-13

**Authors:** Niek R. Hessels, Yinjie Zhu, Stephan J. L. Bakker, Martin H. de Borst, Gerjan J. Navis, Ineke J. Riphagen

**Affiliations:** 1 Department of Internal Medicine, Division of Nephrology, University of Groningen, University Medical Center Groningen, 9713 GZ Groningen, The Netherlands; 2 Department of Laboratory Medicine, University of Groningen, University Medical Center Groningen, 9713 GZ Groningen, The Netherlands; 3 Certe Medical Diagnostics and Advice, 8934 AD Leeuwarden, The Netherlands

**Keywords:** nutrition, preventive medicine, public health, mortality, dietary intake, diet, lifestyle, older persons

## Abstract

Background: Several studies have found a U-shaped association between sodium intake and mortality. The increased mortality risk of low sodium intake has raised debates and hampers widespread acceptance of public health campaigns and dietary guidelines on reducing sodium intake. Whether the excess risk can be attributed to low sodium intake alone or concomitant inadequate intake of other relevant nutrients is unknown. Objective: We investigated whether concomitant low protein intake could explain the lower part of the U-shaped association of sodium intake with all-cause mortality. Methods: We included 1603 individuals aged between 60 and 75 years old from the gender- and socioeconomic status-balanced prospective Lifelines-MINUTHE cohort study. Using multivariable Cox regression analyses, we investigated the association of sodium intake (24 h urinary sodium excretion) with all-cause mortality, including the interaction with protein intake calculated from the Maroni formula. Results: Mean intakes of sodium and protein were 3.9 ± 1.6 g/day and 1.1 ± 0.3 g/kg/day, respectively. After a median follow-up of 8.9 years, 125 individuals (7.8%) had died. The proportion of participants with insufficient protein intake (<0.8 g/kg/day) was inversely related to sodium intake (i.e., 23.3% in Q1 versus 2.8% in Q4, *p* < 0.001). We found an increased risk for mortality in both the highest quartile (Q4, >4.7 g/day; hazard ratio (HR) 1.74 (95% confidence interval (CI) 1.03–2.95)) and the lowest two quartiles of sodium intake (Q1, 0.7–2.8 g/day; 2.05 (1.16–3.62); *p* = 0.01 and Q2, 2.8–3.6 g/day; 1.85 (1.08–3.20); *p* = 0.03), compared with the third quartile of sodium intake (Q3, 3.6–4.7 g/day). This U-shaped association was significantly modified by protein intake (P_interaction_ = 0.006), with the increased mortality risk of low sodium intake being reversed to the lowest mortality risk with concomitant high protein intake. In contrast, the increased mortality risk of low sodium intake was magnified by concomitant low protein intake. Conclusions: We found that a higher protein intake counteracts the increased mortality risk observed in subjects with a low sodium intake. In contrast, a joint low intake of sodium and protein is associated with an increased mortality risk, allegedly due to poor nutritional status. These findings support the guidelines that advocate a lower sodium intake, while highlighting the importance of recognizing overall nutritional status among older adults.

## 1. Introduction

The prevalence of non-communicable diseases (NCD), including hypertension and cardiovascular disease (CVD), has disturbingly increased in recent decades [1,2]. High sodium intake, e.g., due to a high intake of ultra-processed foods, is strongly associated with these outcome measures and is a worldwide public health issue [3,4,5,6,7]. Therefore, the World Health Organization (WHO), the 2015–2020 Dietary Guidelines for Americans, the Dutch Health Council, and other European health organizations advocate a sodium intake below the level of around 2.0 to 2.4 g/day (equal to <5–6 g/day of salt) [1,8,9,10,11,12]. However, in 2013, the Institute of Medicine (IOM) stated that the health effects of sodium intakes below 2.3 g/24 h were inconclusive and uncertain, regarding the association of low sodium intake with cardiovascular events and (all-cause) mortality [13]. Indeed, several observational cohort studies have reported a U- or J-shaped association of sodium intake with CVD and mortality, with sodium intakes of <2.7 g/day and >5 g/day being associated with an increased risk of CVD and mortality [14,15,16,17,18]. In contrast, meta-analyses and other studies that only used 24 h urinary data show that it is likely that studies also using spot urine tests may distort the results and that the association between sodium intake and mortality is linear [15,19,20]. The inconsistent and repeated finding of a J- or U-shaped curve, which at least in part may be due to the use of different methods to quantify sodium intake [21,22], raises questions about the safety of low sodium intake and hampers widespread acceptance of public health campaigns and dietary guidelines.

Whether the association of low sodium intake with increased mortality in observational studies is causally related to low sodium intake or that low sodium intake is a marker for other underlying factors has not been established. Studies from the nephrology field have shown that sodium intake is usually correlated with protein intake [23]. In addition, since the Western diet is known for its richness in sodium and protein [24], a decrease in either protein or sodium content likely leads to a concomitant reduction in the other. For example, an association between dietary sodium and protein intake (24 h urinary data) has been demonstrated in non-diabetic nephrology patients [23] and black hypertensive patients [25]. Furthermore, sodium restriction is linked to a significant reduction in albuminuria and proteinuria in type 2 diabetes (T2D) patients [26] and kidney patients [27] in outpatient settings, respectively. To our knowledge, no data are available on the association between 24 h sodium excretion and protein intake in the general population. Inadequate protein intake can compromise nutritional status, and hence contribute to the mortality risk [28].

These findings suggest that low sodium intake may reflect low protein intake in many subjects, which might be involved in the increased mortality risk associated with low sodium intake [29]. Therefore, we investigated whether the association of low sodium intake and low protein intake was also present in a well-documented subset of elderly (60–75 years) patients stratified based on their socioeconomic status (SES); the Lifelines-MIcroNUTrients and Health inequalities in Elderly (MINUTHE) prospective cohort. Next, we investigated the effect of the interaction between sodium and protein intake on all-cause mortality.

## 2. Methods

### 2.1. Study Design and Population

The Lifelines cohort study is an ongoing observational population-based cohort study that aims to investigate the health and health-related behaviors in aging populations. Between 2006 and 2013, 167,729 residents from the Northern Netherlands were included to form a cohort that was representative of the Dutch general population. A detailed description of the Lifelines cohort study can be found elsewhere [30,31,32]. In short, the first group of participants was recruited by their local general practitioners. These participants could indicate whether their family members were also interested in participating. Individuals willing to participate in the study could register via online self-registration. Individuals with insufficient knowledge of the Dutch language, terminal illness (i.e., life expectancy <5 years), or those with physical or severe psychiatric illness were not eligible to participate. Furthermore, adult participants (≥18 years) were asked to complete several questionnaires regarding demographics, socioeconomic conditions, and lifestyle behaviors.

**Figure 1:**
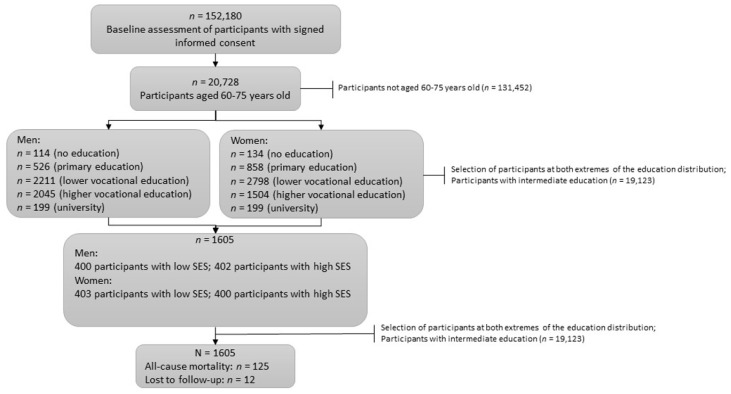
Flow chart of inclusion and exclusion criteria of MINUTHE cohort in this study. Abbreviation: SES, socioeconomic status.

In the current study, a sub-cohort from the Lifelines Biobank, the MINUTHE cohort, was used. This gender- and SES-balanced cohort comprises 1605 participants aged between 60 and 75 years. The MINUTHE cohort was designed to include the following four equal groups: 400 men and 403 women with low SES and 402 men and 400 women with high SES. In a previous study, it was found that SES is more differentiated by education than by income [33]. Hence, SES was based on attained education in this cohort. Low SES was defined as those who had completed, at most, primary school or completed lower vocational or secondary schooling. High SES was defined as those who had completed higher vocational schooling or university education. More details on SES classification are described elsewhere [34]. Two individuals were excluded due to missing data on 24 h urinary sodium excretion specifically, leaving 1603 participants for final analyses (Figure 1).

### 2.2. Exposure Assessments

Fasting blood samples and 24 h urine samples were collected at baseline and transported to the central Lifelines laboratory in Groningen, The Netherlands. Part of the samples was directly transferred to the central laboratory of the University Medical Center Groningen (UMCG) for performing clinical chemistry analyses on fresh blood, plasma, and 24 h urine samples. The remaining samples were stored at −80 °C until analysis. The Maroni formula (i.e., (24 h urea excretion × 0.18) + 15 + 24 h protein excretion)/weight (kg)) was used to estimate the daily protein intake (g/kg/day) [35].

### 2.3. Ascertainment of Outcomes

The outcome of this study was all-cause mortality. Data on mortality were obtained from the municipal register in December 2020.

### 2.4. Lifestyle Factors

Habitual dietary energy, alcohol intake, and protein intake were estimated from a semi-quantitative self-reported food frequency questionnaire (FFQ), using the 2011 Dutch Food Composition Database (NEVO) [36]. The FFQ was developed and validated by Wageningen University to assess the intake of 110 food items over the last month [37,38]. The Lifelines Diet Score (LLDS) was calculated to evaluate the overall diet quality based on the FFQ, which has been described in detail elsewhere [39]. Smoking (never, former smoker and current smoker), sleeping time, and TV watching time (hours/day) were derived from self-administrated questionnaires. The validated Short QUestionnaire to ASsess Health-enhancing physical activity (SQUASH) data were used to calculate the non-occupational moderate-to-vigorous physical activity (MVPA) scores (min/week). These data included information on commuting physical activities and leisure time, including sports, at moderate (4.0–6.4 metabolic equivalent of task (MET)) to vigorous (≥6.5 MET) intensity [40] and were used to calculate the MVPA scores (min/week).

### 2.5. Anthropometric Measurements and Comorbidities

Blood pressure (BP) and anthropometric measurements were performed by well-trained staff. Blood pressure was measured by Dynamap PRO 100 V2 (GE Healthcare, Freiburg, Germany) [32]. Within 10 min, systolic blood pressure (SBP) and diastolic blood pressure were measured ten times in a row. Blood pressure was then calculated as the average of the last three measurements.

Cardiovascular and renal diseases were scored according to the 10th edition of the International Statistical Classification of Diseases and Related Health Problems [41]. In short, hypertension (HT) was defined as BP > 140/90 mmHg or the use of antihypertensive drugs. CVD was considered present if either hypertension, heart failure, atrial fibrillation, or vascular disease was present. Renal disease was defined as the detection of albuminuria (24 h albumin >30 mg) or estimated glomerular filtration rate (eGFR) <60 mL/min/1.73 m^2^. T2D was defined as self-reported T2D (from questionnaires), or the use of oral anti-diabetics and/or the use of insulin or fulfillment of the American Diabetes Association criteria of 2017, which includes a fasting glucose level >6.99 mmol/L or non-fasting glucose level >11.0 mmol/L or hemoglobin A1C (HbA1C) ≥ 6.5% [42].

### 2.6. Statistical Analyses

The baseline characteristics are presented for the total population and according to the quartiles of sodium intake (24 h urinary sodium excretion). Continuous data are presented as the mean ± standard deviation (SD) when normally distributed or as the median and interquartile range (IQR) for abnormally distributed variables. Categorical variables are presented as total numbers (%). ANOVA, Kruskal–Wallis and chi-square tests were performed to determine the between-group differences for the normally distributed continuous data, abnormally distributed continuous data and categorical data, respectively. For the baseline characteristics, three groups were made for estimated daily protein intake, i.e., low (<0.8 g/kg/day), moderate (0.8–1.2 g/kg/day), and high (>1.2 g/kg/day), according to the recommended dietary protein intake [43,44].

We used multivariable Cox regression proportional hazard analysis, using five sequential models to investigate the association between sodium intake and all-cause mortality. Model 1 was adjusted for age. Model 2 was additionally adjusted for SES. Model 3 was adjusted for body mass index (BMI) and the presence of T2D, CVD, or renal disease. Model 4 was adjusted for the LLDS and dietary energy intake. Model 5 was adjusted for other lifestyle factors, i.e., smoking status, TV watching time, sleeping time, MVPA, and alcohol consumption. Scaled Schoenfeld residuals were used to check the proportional hazard assumption. To present the risk of all-cause mortality, equal quartiles of sodium intake were made. As the range of the third quartile (3.6–4.7 g/24 h; mean 3.9 g/24 h) best reflects the intake of >2.3 g/day and <5 g/day, which has been shown to be most beneficial with regard to (CV) mortality [14,15,16,17,18], it was used as the reference group.

We tested for interactions using Cox regression. We fitted an interaction term between sodium intake and protein intake as continuous variable in each of the five sequential models mentioned previously. Three-dimensional estimated hazard ratios were plotted for each observed pair of sodium intake and protein intake in the analysis dataset, using the R package “plot3D” (R Core Team, Vienna, Austria) to visualize the change in hazards for the interaction between sodium and protein intake.

Calculated protein intake using the Maroni formula is not widely used in clinical practice yet and can also be influenced by body composition. Therefore, we also tested for an interaction between sodium intake and other clinical markers for protein intake, including 24 h urinary urea excretion and BMI-adjusted estimated protein intake, respectively. The BMI-adjusted estimated protein intake was adjusted for underweight and obese participants to avoid potential bias; a BMI <20 kg/m^2^ and a BMI ≥30 kg/m^2^ were adjusted to 20 kg/m^2^ and 27.5 kg/m^2^, respectively [45].

All analyses were carried out using RStudio (version 1.1.463; RStudio Inc., Boston, MA, USA).

### 2.7. Declaration of Helsinki, Informed Consent, and Ethical Approval

Informed consent was obtained from all subjects involved in the study. The study was conducted in accordance with the Declaration of Helsinki, and the protocol was approved by the Medical Ethics committee of the University Medical Center Groningen Institutional Review Board in the Netherlands (METc approval number: 2007/152).

### 2.8. Patient and Public Involvement 

The patients and/or the public were not involved in the design of this study. 

## 3. Results

In total, 1603 individuals of the Lifelines-MINUTHE cohort, aged between 60 and 75 years old, were included in this study (Figure 1). After a median follow-up period of 8.9 (IQR 7.9–10.1) years, 125 individuals had died (7.8%). The baseline characteristics are presented in Table 1 for the total population and quartiles of sodium intake. In the total population, mean sodium and mean estimated protein intake were 3.9 ± 1.6 g/day and 1.1 ± 0.3 g/kg/day, respectively. Interestingly, the proportion of participants with insufficient protein intake (<0.8 g/kg/day) was inversely related to sodium intake (i.e., 23.3% in Q1, as opposed to 2.8% in Q4, *p* < 0.001). Moreover, sodium intake was associated with several lifestyle factors, including the Lifelines Diet Score, which was inversely associated with sodium intake.

### 3.1. Sodium intake and All-Cause Mortality

Compared to a sodium intake of 3.6–4.7 g/day (Q3, the reference group), both high sodium intake (Q4; hazard ratio (HR) 1.74 (95% confidence interval (CI) 1.03–2.95); *p* = 0.04) and the two lower quartiles of sodium intake (Q1; 2.05 (1.16–3.62); *p* = 0.01 and Q2; 1.85 (1.08–3.20); *p* = 0.03) were associated with an increased risk of all-cause mortality in the multivariable analyses (Table 2), with associations independent of the adjustment for potential confounders.

### 3.2. Effect of Interaction between Sodium Intake and Protein Intake on Mortality

**Figure 2:**
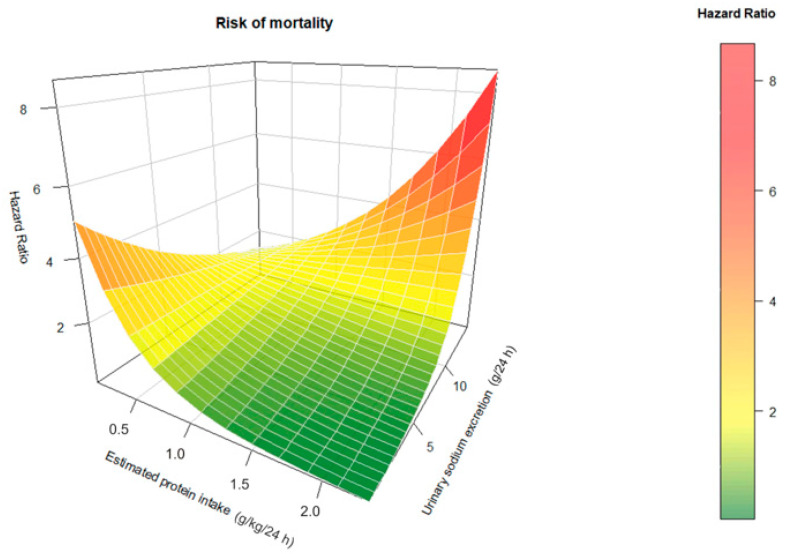
Three-dimensional graph of the combined effect of sodium intake and protein intake calculated by the Maroni formula with all-cause mortality.

We found a significant interaction between sodium intake and protein intake regarding their association with mortality. This interaction was present in all the multivariable models (e.g., model 1, *p* = 0.006; model 5, *p* = 0.01; Table 3). Figure 2 shows the 3D surface plot of the joint association between sodium intake and protein intake and all-cause mortality. In the subjects with low sodium intake, the mortality risk was higher when protein intake was also low. In contrast, the increased mortality risk, otherwise caused by low sodium intake alone, was inverted to the lowest mortality risk when protein intake was higher.

### 3.3. Sensitivity Analyses

In the sensitivity analyses, in which either 24 h urea excretion (Supplementary Materials Figure S1) or BMI-adjusted protein intake (Supplementary Materials Figure S2) was used instead of protein intake calculated by the Maroni formula, similar joint effects were found.

## 4. Discussion

In this study, high and low sodium intake were associated with higher all-cause mortality risks, which is similar to previous literature findings. In line with prior studies in smaller populations, we found that sodium intake and protein intake were associated with an overrepresentation of inadequate protein intake among those with the lowest sodium intake. We found a significant interaction effect between sodium intake and protein intake on all-cause mortality, with the lowest all-cause mortality risk in individuals with a low sodium intake combined with a higher protein intake. In contrast, the all-cause mortality risk was the highest in individuals in whom both their sodium and protein intake were low. These findings were independent of potential confounders, including lifestyle factors and diet quality assessed by the LLDS. Therefore, these data suggest that the previously described excess mortality in the lower part of the U-shaped curve is the consequence of concomitant lower protein intake, either through lower protein intake itself or by lower protein intake being a proxy for poor overall nutritional status.

### 4.1. Comparisons with Other Studies

The U-shaped relationship of sodium intake with all-cause mortality found in this study has been reported previously [13,14,15,46]. It has led to controversy in the field, as it contrasts with the current public health policy advocated by the WHO and other international health organizations regarding lower sodium intake [1,8,9,10,11,12]. The true existence of a U-shaped association between sodium intake, CV- and all-cause) mortality has been questioned. Most studies that describe a J- or U-shaped relationship between sodium intake and CVD, CV mortality or all-cause mortality use spot urine or overnight urine samples (13,14,16), which might have distorted the results among other possible confounders [22,47,48]. For example, a large meta-analysis by Wang et al. found a linear and dose-dependent association between 24 h sodium excretion and CV risk [19]. In addition, a systematic analysis of the Global Burden of Disease study showed that, when looking at the dietary risk factors that contribute to CV mortality, higher 24 h sodium excretion was associated with higher CV mortality, and a sodium reduction resulted in lower CV mortality [20]. Given this uncertainty, studying other conflicting results regarding sodium intake and mortality could provide substantial evidence on this topic.

To the best of our knowledge, there are no studies that have reported the effect of protein intake on the association between 24 h urinary sodium excretion and mortality in the general population. Previous studies from our group showed a consistent association between sodium consumption and protein intake in non-diabetic kidney patients [23], possibly due to the combined presence of sodium and protein in many of the food products in the Western diet [24]. In this randomized controlled trial (RCT), which included 52 patients with non-diabetic nephropathy, the dietary protein intake assessed through the Maroni formula changed significantly from 1.02 ± 0.04 g/kg/day during angiotensin-converting enzyme (ACE) inhibition and following a regular sodium diet (200 mmol Na^+^/day) to 0.96 ± 0.04 g/kg/day (*p* = 0.004) on a low sodium diet (160 mmol Na^+^/day) [23]. In addition, Swift et al. found that a reduction in 24 h sodium excretion from 169 ± 73 to 89 ± 52 mmol/24 h (*p* < 0.001) resulted in a reduction in protein excretion from 93 ± 48 mg to 75 ± 30 mg/24 h (*p* < 0.008) in 40 black hypertensive patients included in a double-blind RCT [25]. Moreover, sodium restriction is linked to a significant reduction in albuminuria and proteinuria in T2D patients [26] and kidney patients [27] in outpatient settings, respectively. In conclusion, although relevant data are scarce, successful sodium restriction seems to elicit a small, but significant, reduction in protein intake in clinical practice, which aligns with our findings in the present study.

Therefore, dietary counseling in patients and the overall population aims to reduce sodium intake, while striving for an adequate overall diet [49,50,51]. Our current data show that the overall association between sodium and protein intake is also present in the elderly members of the general population. Such an observation has not been made before in cohorts from the general population. However, the relevance of adequate protein intake for nutritional status was shown in heart failure patients in the Third National Health and Nutrition Examination Survey (NHANES III) study [52]. In these patients, where sodium restriction was an integral part of the treatment, sodium intake corresponded to the lower sodium quartiles in our study. In addition, in heart failure patients from the Biology Study to Tailored Treatment in Chronic Heart Failure (BIOSTAT-CHF) trial, a higher protein intake (70+ g/day vs. ≤40 g/day) was associated with substantially lower mortality rates (18 vs. 32%; *p* < 0.001) [53], supporting the role of adequate protein intake in vulnerable populations, albeit not demonstrating the cause or consequence. This information underlines the relevance of measuring other dietary factors in the relationship between sodium and clinical outcome, such as mortality. Lastly, we measured overall diet quality, expressed as the LLDS, as well as energy intake and other lifestyle factors, to account for their possible contribution to the outcome. However, neither of these factors affected our current results.

### 4.2. Implications for Clinicians and Policymakers

International dietary guidelines and the WHO fact sheets regarding healthy diets are regularly updated to improve the feasibility of the improvement of global health. The U-shaped curve between sodium intake and all-cause mortality has led to controversy in the field. However, it is likely to be biased by the underlying effects of (protein) malnutrition. Overall, the protein intakes in our cohort are consistent with the numbers reported in older adults [54]. Of note, one out of four (23%) individuals in the lowest sodium quartile consumed <0.8 g/kg/day of protein, i.e., the lowest recommended dietary allowance (RDA) of protein for a healthy adult with minimal physical activity [44,55]. Although the recommendations on protein intake for the elderly are inconsistent, an intake of 0.8 g/kg/day is invariably considered to be inadequate.

In light of the results found in this study, in which a concomitant low intake of sodium and protein increases the risk of all-cause mortality, proper nutritional status with sufficient protein intake could be considered as a prerequisite for reducing sodium intake. Overall, our findings suggest nutritional status should receive more attention in public health campaigns, while also reconfirming the fact that it is crucial to not only look at sodium as an isolated phenomenon, but to look at food patterns.

### 4.3. Strengths and Weaknesses of This Study

The major strengths of the present study are the use of objective measurements of sodium and urea with 24 h urine samples and the extensive documentation of diet and lifestyle. The “gold standard” 24 h sodium specimens [56] used in this study are less prone to errors than the estimated sodium intake values obtained through overnight or spot urine samples. In addition, calculating protein intake based on the estimation of the nitrogen balance in patients is considered as the gold standard for protein intake assessment [57,58].

However, several limitations must be noted for this study. First, only data on baseline sodium intake were available. This could lead to regression dilution bias [47]. This limitation is shared with most observational analyses, in which excretion data are linked to long-term outcome data.

Second, we cannot exclude the possibility of reverse causation. Individuals with comorbidities such as T2D and CVD might have been advised on sodium intake, and those with renal disease may have been advised on protein intake [59]. However, in this study, the prevalence of renal disease was low, and it is unlikely that reverse causation may have elicited the interaction between sodium and protein intake. In addition, we attempted to mitigate these effects by adjusting for these comorbidities in our analyses.

Third, we studied older adults aged between 60 and 75 years old from the Northern Netherlands, in a predominantly Caucasian population, in whom the Western diet dominates their dietary habits, hampering generalizability to populations with other ethnicities and other dietary habits, and renal and cardiovascular patients.

Fourth, the present study is based on all-cause mortality, not CV mortality. A recent meta-analysis found that dietary risks accounted for 49.2% of cardiovascular deaths and 22.4% of all-cause deaths, making CV mortality the preferable outcome [20]. Unfortunately, CV mortality data were unavailable in this study population.

Fifth, we used the LLDS, which is based on self-reported FFQ data, to adjust for diet quality. The self-reported FFQ data could potentially impact the quality of the dietary data collected for this study. Therefore, the LLDS was developed based on diet–disease relationships and corresponded to the Dutch dietary guidelines in the Lifelines population. After development, the LLDS has been found to be a reliable tool for dietary quality assessment [39,60].

Lastly, an observational study such as this always bears the risk of residual confounding. Therefore, all major confounders of the effect of the association between sodium and protein intake on all-cause mortality were included in the analyses to limit this effect.

## 5. Conclusions

Our findings indicate that a higher protein intake counteracts the excess mortality observed in subjects with a low sodium intake, whereas a joint low intake of sodium and protein is associated with an exceptionally high mortality risk, allegedly due to poor nutritional status. The lowest mortality risk was found in individuals with a low sodium and higher protein intake. These findings refute the claim that low sodium intake is dangerous, countering one of the contra-arguments to the current public health policy advocated by the WHO and other international health organizations for individuals who consume a Western diet. Overall, our findings support the guidelines that advocate a lower sodium intake and add to the notion that future guidelines should consider the importance of overall nutritional status when a low-sodium diet is advocated.

## Figures and Tables

**Table 1:** Baseline characteristics of the MINUTHE cohort for total population and population distributed by equal sodium intake quartiles.

	Total (*n* = 1603)	Quartiles of Sodium Intake				
		Q1 (*n* = 401)	Q2 (*n* = 401)	Q3 (*n* = 401)	Q4 (*n* = 400)	*p*
Na, g/day (range)		0.7–2.8	2.8–3.6	3.6–4.7	4.7–14.3	
						
Demographics						
Male, %	50	24.9	42.4	55.9	76.7	<0.001
Age, years	66 ± 4	66 ± 4	66 ± 4	66 ± 4	65 ± 4	0.7
Low SES, %	50	45.1	45.1	50.6	59.2	<0.001
Smoking						
Current	12	12.9	10.8	13	11.4	<0.001
Former	53.6	45.1	53.9	53.4	62	
Never	34.4	42.1	35.3	33.6	26.6	
Alcohol, g/day	6.4 (1.2–16)	3.8 (0.8–12)	6.4 (0.8–17)	6.8 (1.7–17)	6.6 (1.6–17)	0.008
Energy intake, kcal/day	1909 ± 518	1777 ± 450	1861 ± 467	1987 ± 549	2011 ± 565	<0.001
MVPA, min/week	260 (110–523)	270 (120–510)	240 (100–500)	300 (120–535)	240 (90–540)	0.6
LLDS	23.9 ± 6.2	25.4 ± 6.2	24.1 ± 6.2	23.3 ± 6.0	22.9 ± 6.0	<0.001
TV watching, h/day	2.9 ± 1.6	2.8 ± 1.5	2.8 ± 1.5	3.1 ± 2	3.0 ± 1.5	0.02
Sleeping, h/day	7.5 ± 1.0	7.6 ± 1.0	7.5 ± 0.9	7.6 ± 1.1	7.4 ± 1.0	0.08
Morbidities						
T2D, %	29	29.4	28.2	28.4	30	0.9
CVD, %	40.9	37.9	38.2	42.9	44.5	0.1
Renal disease, %	5.2	7.2	5.5	4.5	3.5	0.1
Protein intake, g/day	71.8 ± 18.3	66.0 ± 16.0	70.0 ± 17.3	74.2 ± 18.7	77.2 ± 19.4	<0.001
Protein intake, g/kg/day	1.1 ± 0.3	1.0 ± 0.2	1.1 ± 0.2	1.1 ± 0.2	1.2 ± 0.3	<0.001
<0.8	10.3	23.2	10.0	5.0	2.8	<0.001
0.8–1.2	58.6	60.5	64.7	61.3	47.9	
>1.2	31.1	16.2	25.2	33.7	49.4	
BMI, kg/m^2^	26.9 ± 4.1	26.1 ± 4.3	26.5 ± 3.9	26.7 ± 4.0	28.4 ± 3.8	<0.001
SBP, mmHg	134 ± 17	132 ± 18	134 ± 18	135 ± 17	135 ± 16	0.06
DBP, mmHg	75 ± 9	74 ± 9	75 ± 9	76 ± 9	76 ± 9	0.004
						
Urinary parameters						
Na intake, g/day	3.9 ± 1.6	2.2 ± 0.5	3.2 ± 0.2	4.1 ± 0.3	6.0 ± 1.3	<0.001
K excretion, g/day	3.5 ± 1.1	2.9 ± 0.9	3.3 ± 0.9	3.6 ± 1.0	4.2 ± 1.1	<0.001
Na/K ratio	1.8 (1.4–2.3)	1.3 (1.0–1.6)	1.6 (1.3–2.0)	1.9 (1.6–2.3)	2.3 (2.0–2.8)	<0.001
Total protein excretion, g/24 h	0.11 (0.10–0.15)	0.1 (0.09–0.12)	0.11 (0.10–0.14)	0.12 (0.11–0.14)	0.14 (0.12–0.17)	<0.001
Urea, mmol/24 h	399 ± 129	302 ± 89	372 ± 94	408 ± 95	515 ± 131	<0.001
Creatinine, mmol/24 h	12.0 ± 3.8	9.4 ± 2.8	11.2 ± 2.9	12.4 ± 3.0	15.2 ± 3.8	<0.001

Values are mean ± standard deviation and median (interquartile range) for continuous and categoric variables or numbers (percentage), respectively. Abbreviations: Q1–4, sodium intake (24 h urinary sodium excretion)-based quartiles from lowest intake through to highest intake; T2D: type 2 diabetes; CVD, cardiovascular disease; SES, socioeconomic status; MVPA, moderate-to-vigorous physical activity; LLDS, Lifelines Diet Score; BMI, body mass index; SBP, systolic blood pressure; DBP, diastolic blood pressure; Na, sodium; K, potassium; Na/K ratio, sodium-to-potassium ratio.

**Table 2:** All-cause mortality for equal quartiles of sodium intake in the MINUTHE cohort, with the third quartile as the reference group.

	Q1 (0.7–2.8 g/day)		Q2 (2.8–3.6 g/day)		Q3 (3.6–4.7 g/day)	Q4 (4.7–14.3 g/day)		
	HR (95% CI)	*p*-Value	HR (95% CI)	*p*-Value	Reference	HR (95% CI)	*p*-Value	*p*-Trend
Model 1	2.05 (1.16–3.62)	0.01	1.85 (1.08–3.20)	0.03	1	1.74 (1.03–2.95)	0.04	0.07
Model 2	2.12 (1.20–3.74)	0.01	1.89 (1.09–3.25)	0.02	1	1.68 (0.99–2.84)	0.06	0.09
Model 3	2.05 (1.16–3.62)	0.01	1.85 (1.08–3.19)	0.03	1	1.75 (1.03–2.98)	0.04	0.06
Model 4	1.97 (1.11–3.48)	0.02	1.82 (1.06–3.14)	0.03	1	1.76 (1.03–3.00)	0.04	0.05
Model 5	1.94 (1.09–3.47)	0.03	1.79 (1.03–3.09)	0.04	1	1.70 (1.00–2.91)	0.05	0.07

Model 1: adjusted for age and sex. Model 2: model 1 + SES. Model 3: model 2 + BMI, and presence of type 2 diabetes and cardiovascular and renal disease. Model 4: model 3 + dietary factors (LLDS + total energy intake). Model 5: model 4 + other lifestyle factors (smoking status, TV watching time, sleeping time, alcohol consumption, and MVPA). Abbreviations: Q1–4, sodium intake (24 h urinary sodium excretion)-based quartiles from lowest intake through to highest intake; HR, hazard ratio; 95% CI, 95% confidence interval; SES, socioeconomic status; BMI, body mass index; LLDS, Lifelines Diet Score; MVPA, moderate-to-vigorous physical activity.

**Table 3:** Associations between sodium intake, protein intake, and their interaction term with all-cause mortality.

	Model 1		Model 2		Model 3		Model 4		Model 5	
	HR (95% CI)	*p*-Value	HR (95% CI)	*p*-Value	HR (95% CI)	*p*-Value	HR (95% CI)	*p*-Value	HR (95% CI)	*p*-Value
Sodium intake, g/day	0.71 (0.54–0.95)	0.02	0.70 (0.52–0.93)	0.02	0.72 (0.53–0.98)	0.04	0.74 (0.54–1.01)	0.05	0.71 (0.52–0.97)	0.04
Protein intake, g/day	0.10 (0.03–0.32)	<0.001	0.11 (0.03–0.37)	<0.001	0.09 (0.02–0.32)	<0.001	0.10 (0.03–0.37)	0.001	0.10 (0.02–0.39)	0.001
Sodium intake × protein intake	1.34 (1.09–1.65)	0.006	1.34 (1.09–1.64)	0.007	1.34 (1.08–1.68)	0.01	1.33 (1.06–1.66)	0.02	1.35 (1.07–1.69)	0.01

Model 1: adjusted for age and sex. Model 2: model 1 + SES. Model 3: model 2 + BMI, and presence of type 2 diabetes and cardiovascular and renal disease. Model 4: model 3 + dietary factors (LLDS + total energy intake). Model 5: model 4 + other lifestyle factors (smoking status, TV watching time, sleeping time, alcohol consumption, and MVPA). Abbreviations: HR, hazard ratio; 95% CI, 95% confidence interval; SES, socioeconomic status; BMI, body mass index; LLDS, Lifelines Diet Score; MVPA, moderate-to-vigorous physical activity.

## Data Availability

The Lifelines biobank is an international resource for health research. Information on data access and application is available on www.lifelines.nl.

## Supplementary Materials

The following supporting information can be downloaded at: https://www.mdpi.com/article/10.3390/nu15020428/s1, Figure S1: Joint association of 24 h sodium intake and urea excretion with all-cause mortality; Figure S2: Joint association of 24 h sodium intake and BMI adjusted protein intake with all-cause mortality.

## References

[1] Powles J., Fahimi S., Micha R., Khatibzadeh S., Shi P., Ezzati M., Engell R.E., Lim S.S., Danaei G., Mozaffarian D. (2013). Global, regional and national sodium intakes in 1990 and 2010: A systematic analysis of 24 h urinary sodium excretion and dietary surveys worldwide. BMJ Open.

[2] Thout S.R., Santos J.A., McKenzie B., Trieu K., Johnson C., McLean R., Arcand J.A., Campbell N.R.C., Webster J. (2019). The Science of Salt: Updating the evidence on global estimates of salt intake. J. Clin. Hypertens..

[3] Weinberger M.H. (1996). Salt sensitivity of blood pressure in humans. Hypertens.

[4] Strazzullo P., D’Elia L., Kandala N.-B., Cappuccio F.P. (2009). Salt intake, stroke, and cardiovascular disease: Meta-analysis of prospective studies. BMJ.

[5] Olde Engberink R.H.G., Van Den Hoek T.C., Van Noordenne N.D., Van Den Born B.J.H., Peters-Sengers H., Vogt L. (2017). Use of a single baseline versus multiyear 24-hour urine collection for estimation of long-term sodium intake and associated cardiovascular and renal risk. Circulation.

[6] Cogswell M.E., Mugavero K., Bowman B.A., Frieden T.R. (2016). Dietary Sodium and Cardiovascular Disease Risk–Measurement Matters. N. Engl. J. Med..

[7] He F.J., Marciniak M., Visagie E., Markandu N.D., Anand V., Dalton R.N., MacGregor G.A. (2009). Effect of modest salt reduction on blood pressure, urinary albumin, and pulse wave velocity in white, black, and asian mild hypertensives. Hypertension.

[8] World Health Organization (WHO) (2013). Prevention of Recurrent Heart Attacks and Strokes in Low and Middle Income Populations: Evidence-Based Recommendations for Policy Makers and Health Professionals.

[9] Turck D., Castenmiller J., de Henauw S., Hirsch-Ernst K.I., Kearney J., Knutsen H.K., Maciuk A., Mangelsdorf I., McArdle H.J., Pelaez C. (2019). Dietary reference values for sodium. EFSA J..

[10] Strohm D., Bechthold A., Ellinger S., Leschik-Bonnet E., Stehle P., Heseker H. (2018). Revised reference values for the intake of sodium and chloride. Ann. Nutr. Metab..

[11] U.S. Department of Health and Human Services, U.S. Department of Agriculture (2015). 2015–2020 Dietary Guidelines for Americans.

[12] Nordic Nutrition Recommendations (2014). Nordic Nutrition Recommendations 2012: Integrating Nutrition and Physical Activity.

[13] McGuire S. (2014). Institute of Medicine. 2013. “Sodium intake in populations: Assessment of evidence.” The National Academies Press: Washington, DC, USA, 2013. Adv. Nutr..

[14] O’Donnell M., Mente A., Rangarajan S., McQueen M.J., Wang X., Liu L., Yan H., Lee S.F., Mony P., Devanath A. (2014). Urinary Sodium and Potassium Excretion, Mortality, and Cardiovascular Events. N. Engl. J. Med..

[15] O’Donnell M., Mente A., Rangarajan S., McQueen M.J., O’Leary N., Yin L., Liu X., Swaminathan S., Khatib R., Rosengren A. (2019). Joint association of urinary sodium and potassium excretion with cardiovascular events and mortality: Prospective cohort study. BMJ.

[16] Graudal N., Jürgens G., Baslund B., Alderman M.H. (2014). Compared with usual sodium intake, low- and excessive-sodium diets are associated with increased mortality: A meta-analysis. Am. J. Hypertens..

[17] Pfister R., Michels G., Sharp S.J., Luben R., Wareham N.J., Khaw K.T. (2014). Estimated urinary sodium excretion and risk of heart failure in men and women in the EPIC-Norfolk study. Eur. J. Heart Fail..

[18] Ekinci E.I., Clarke S., Thomas M.C., Moran J.L., Cheong K., MacIsaac R.J., Jerums G. (2011). Dietary salt intake and mortality in patients with type 2 diabetes. Diabetes Care.

[19] Wang Y.-J., Yeh T.-L., Shih M.-C., Tu Y.-K., Chien K.-L. (2020). Dietary Sodium Intake and Risk of Cardiovascular Disease: A Systematic Review and Dose-Response Meta-Analysis. Nutrients.

[20] Meier T., Gräfe K., Senn F., Sur P., Stangl G.I., Dawczynski C., März W., Kleber M.E., Lorkowski S. (2019). Cardiovascular mortality attributable to dietary risk factors in 51 countries in the WHO European Region from 1990 to 2016: A systematic analysis of the Global Burden of Disease Study. Eur. J. Epidemiol..

[21] O’Donnell M.J., Mente A., Smyth A., Yusuf S. (2013). Salt intake and cardiovascular disease: Why are the data inconsistent?. Eur. Heart J..

[22] O’Donnell M., Mente A., Alderman M.H., Brady A.J.B., Diaz R., Gupta R., López-Jaramillo P., Luft F.C., Lüscher T.F., Mancia G. (2020). Salt and cardiovascular disease: Insufficient evidence to recommend low sodium intake. Eur. Heart J..

[23] Slagman M.C.J., Waanders F., Hemmelder M.H., Woittiez A.-J., Janssen W.M.T., Lambers Heerspink H.J., Navis G., Laverman G.D. (2011). Moderate dietary sodium restriction added to angiotensin converting enzyme inhibition compared with dual blockade in lowering proteinuria and blood pressure: Randomised controlled trial. BMJ.

[24] Odermatt A. (2011). The western-style diet: A major risk factor for impaired kidney function and chronic kidney disease. Am. J. Physiol.-Ren. Physiol..

[25] Swift P.A., Markandu N.D., Sagnella G.A., He F.J., MacGregor G.A. (2005). Modest salt reduction reduces blood pressure and urine protein excretion in black hypertensives: A randomized control trial. Hypertension.

[26] Suckling R.J., He F.J., Markandu N.D., Macgregor G.A. (2016). Modest salt reduction lowers blood pressure and albumin excretion in impaired glucose tolerance and type 2 diabetes mellitus: A randomized double-blind trial. Hypertension.

[27] McMahon E.J., Campbell K.L., Bauer J.D., Mudge D.W. (2015). Altered dietary salt intake for people with chronic kidney disease. Cochrane database Syst. Rev..

[28] Lorenzo-López L., Maseda A., De Labra C., Regueiro-Folgueira L., Rodríguez-Villamil J.L., Millán-Calenti J.C. (2017). Nutritional determinants of frailty in older adults: A systematic review. BMC Geriatr..

[29] Lambers Heerspink H.J., Kwakernaak A., De Zeeuw D., Navis G. (2011). Comment on: Ekinci et al. Dietary salt intake and mortality in patients with type 2 diabetes. Diabetes Care 2011;34: 703–709. Diabetes Care.

[30] Klijs B., Scholtens S., Mandemakers J.J., Snieder H., Stolk R.P., Smidt N. (2015). Representativeness of the LifeLines cohort study. PLoS ONE.

[31] van der Ende M.Y., Hartman M.H.T., Hagemeijer Y., Meems L.M.G., de Vries H.S., Stolk R.P., de Boer R.A., Sijtsma A., van der Meer P., Rienstra M. (2017). The LifeLines Cohort Study: Prevalence and treatment of cardiovascular disease and risk factors. Int. J. Cardiol..

[32] Scholtens S., Smidt N., Swertz M.A., Bakker S.J.L., Dotinga A., Vonk J.M., Van Dijk F., Van Zon S.K.R., Wijmenga C., Wolffenbuttel B.H.R. (2015). Cohort Profile: LifeLines, a three-generation cohort study and biobank. Int. J. Epidemiol..

[33] Vart P., Gansevoort R.T., Coresh J., Reijneveld S.A., Bültmann U. (2013). Socioeconomic measures and CKD in the United States and The Netherlands. Clin. J. Am. Soc. Nephrol..

[34] Riphagen I.J., Minović I., Groothof D., Post A., Eggersdorfer M.L., Kootstra-Ros J.E., de Borst M.H., Navis G., Muskiet F.A.J., Kema I.P. (2020). Methylmalonic acid, vitamin B12, renal function, and risk of all-cause mortality in the general population: Results from the prospective Lifelines-MINUTHE study. BMC Med..

[35] Maroni B.J., Steinman T.I., Mitch W.E. (1985). A method for estimating nitrogen intake of patients with chronic renal failure. Kidney Int..

[36] (2011). RIVM Dutch food composition table (NEVO). https://www.rivm.nl/nieuws/nieuwe-nevo-tabel-2011-beschikbaar.

[37] Molag M.L., De Vries J.H.M., Duif N., Ocké M.C., Dagnelie P.C., Goldbohm R.A., Van’T Veer P. (2010). Selecting informative food items for compiling food-frequency questionnaires: Comparison of procedures. Br. J. Nutr..

[38] Siebelink E., Geelen A., De Vries J.H.M. (2011). Self-reported energy intake by FFQ compared with actual energy intake to maintain body weight in 516 adults. Br. J. Nutr..

[39] Vinke P.C., Corpeleijn E., Dekker L.H., Jacobs D.R., Navis G., Kromhout D. (2018). Development of the food-based Lifelines Diet Score (LLDS) and its application in 129,369 Lifelines participants. Eur. J. Clin. Nutr..

[40] Wendel-Vos G.C.W., Schuit A.J., Saris W.H.M., Kromhout D. (2003). Reproducibility and relative validity of the short questionnaire to assess health-enhancing physical activity. J. Clin. Epidemiol..

[41] World Health Organization (2010). International Statistical Classification of Diseases and Related Health Problems.

[42] Marathe P.H., Gao H.X., Close K.L. (2017). American Diabetes Association Standards of Medical Care in Diabetes 2017. J. Diabetes.

[43] Trumbo P., Schlicker S., Yates A.A., Poos M. (2002). Dietary reference intakes for energy, carbohydrate, fiber, fat, fatty acids, cholesterol, protein and amino acids. J. Am. Diet. Assoc..

[44] Richter M., Baerlocher K., Bauer J.M., Elmadfa I., Heseker H., Leschik-Bonnet E., Stangl G., Volkert D., Stehle P. (2019). Revised Reference Values for the Intake of Protein. Ann. Nutr. Metab..

[45] Gomes Neto A.W., Boslooper-Meulenbelt K., Geelink M., van Vliet I.M.Y., Post A., Joustra M.L., Knoop H., Berger S.P., Navis G.J., Bakker S.J.L. (2020). Protein intake, fatigue and quality of life in stable outpatient kidney transplant recipients. Nutrients.

[46] Cook N.R., Appel L.J., Whelton P.K. (2016). Sodium Intake and All-Cause Mortality Over 20 Years in the Trials of Hypertension Prevention. J. Am. Coll. Cardiol..

[47] Ma Y., He F.J., Sun Q., Yuan C., Kieneker L.M., Curhan G.C., MacGregor G.A., Bakker S.J.L., Campbell N.R.C., Wang M. (2021). 24-Hour Urinary Sodium and Potassium Excretion and Cardiovascular Risk. N. Engl. J. Med..

[48] Cappuccio F.P., Campbell N.R.C., He F.J., Jacobson M.F., MacGregor G.A., Antman E., Appel L.J., Arcand J.A., Blanco-Metzler A., Cook N.R. (2022). Sodium and Health: Old Myths and a Controversy Based on Denial. Curr. Nutr. Rep..

[49] World Health Organization (2003). Food Based Dietary Guidelines in the WHO European Region, Eur/03/5045414.

[50] EFSA (2010). Scientific Opinion on establishing Food-Based Dietary Guidelines. EFSA J..

[51] World Health Organization (2012). Promoting a Healthy Diet for the WHO Eastern Mediterranean Region: User-Friendly Guide.

[52] Sattler E.L.P., Ishikawa Y., Trivedi-Kapoor R., Zhang D., Quyyumi A.A., Dunbar S.B. (2019). Association between the prognostic nutritional index and dietary intake in community-dwelling older adults with heart failure: Findings from NHANES III. Nutrients.

[53] Streng K.W., Hillege H.L., Maaten J.M., Veldhuisen D.J., Dickstein K., Ng L.L., Samani N.J., Metra M., Ponikowski P., Cleland J.G. (2022). Clinical implications of low estimated protein intake in patients with heart failure. J. Cachexia. Sarcopenia Muscle.

[54] Saka B., Kaya O., Ozturk G.B., Erten N., Karan M.A. (2010). Malnutrition in the elderly and its relationship with other geriatric syndromes. Clin. Nutr..

[55] Rand W.M., Pellett P.L., Young V.R. (2003). Meta-analysis of nitrogen balance studies for estimating protein requirements in healthy adults. Am. J. Clin. Nutr..

[56] Ji C., Sykes L., Paul C., Dary O., Legetic B., Campbell N.R., Cappuccio F.P. (2012). Systematic review of studies comparing 24-hour and spot urine collections for estimating population salt intake. Rev. Panam. Salud Publica/Pan Am. J. Public Heal..

[57] Mitch W.E. (1991). Dietary protein restriction in patients with chronic renal failure. Kidney Int..

[58] Matsuda T., Kato H., Suzuki H., Mizugaki A., Ezaki T., Ogita F. (2018). Within-Day Amino Acid Intakes and Nitrogen Balance in Male Collegiate Swimmers during the General Preparation Phase. Nutrients.

[59] Cobb L.K., Anderson C.A.M., Elliott P., Hu F.B., Liu K., Neaton J.D., Whelton P.K., Woodward M., Appel L.J. (2014). Methodological issues in cohort studies that relate sodium intake to cardiovascular disease outcomes: A science advisory from the American Heart Association. Circulation.

[60] Dekker L.H., De Borst M.H., Meems L.M.G., De Boer R.A., Bakker S.J.L., Navis G.J. (2019). The association of multimorbidity within cardio-metabolic disease domains with dietary patterns: A cross-sectional study in 129 369 men and women from the Lifelines cohort. PLoS ONE.

