# Supplementary material for: Low Sodium Intake, Low Protein Intake, and Excess Mortality in an Older Dutch General Population Cohort: Findings in the Prospective Lifelines-MINUTHE Study
Source: Nutrients. 2023 Jan 13;15(2):428. doi: 10.3390/nu15020428 (PMC9865839; doi:10.3390/nu15020428)
Supplement: Supplementary file 1 [file nutrients-15-00428-s001.zip › nutrients-2100624-supplementary.pdf]

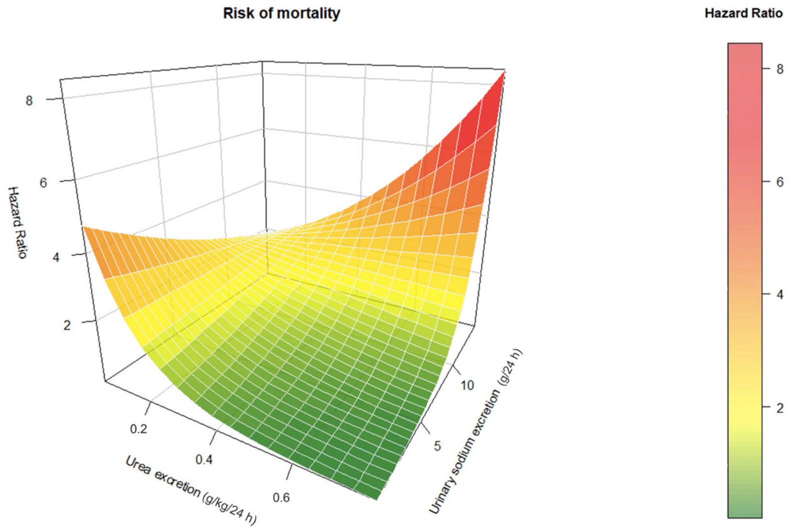

Figure S1. Joint association of 24 h sodium intake and urea excretion with all-cause mortality.

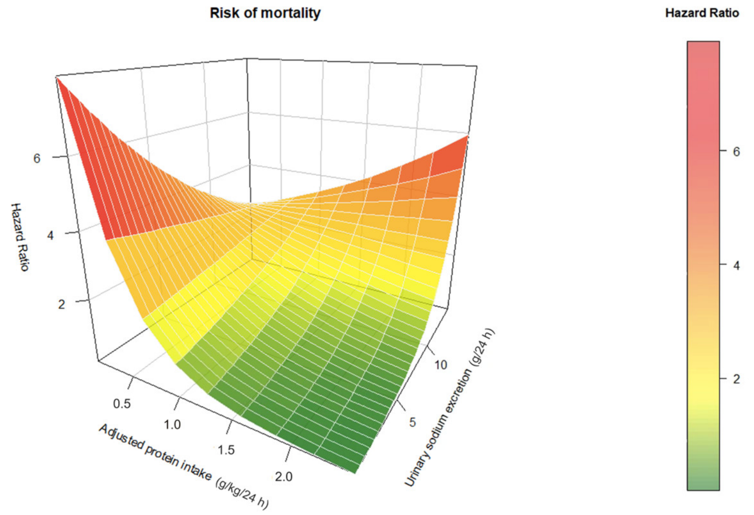

Figure S2. Joint association of 24 h sodium intake and BMI adjusted protein intake with all-cause mortality. Abbreviation: BMI, body mass index.
